# Attenuation of Age-Related Hearing Impairment in Senescence-Accelerated Mouse Prone 8 (SAMP8) Mice Treated with Fatty Acid Synthase Inhibitor CMS121

**DOI:** 10.1007/s12031-023-02119-w

**Published:** 2023-04-25

**Authors:** Tammy B. Pham, Ely Cheikh Boussaty, Antonio Currais, Pamela Maher, David R. Schubert, Uri Manor, Rick A. Friedman

**Affiliations:** 1 Department of Otolaryngology-Head and Neck Surgery, University of California San Diego Health, 92037 La Jolla, CA USA; 2 Cellular Neurobiology Laboratory, Salk Institute for Biological Studies, 92037 La Jolla, CA USA; 3 Waitt Advanced Biophotonics Center, Salk Institute for Biological Studies, 92037 La Jolla, CA USA

**Keywords:** Age-related hearing impairment, Hearing loss, SAMP8, ABR, CMS121

## Abstract

In the senescence-accelerated mouse prone 8 (SAMP8) mouse model, oxidative stress leads to premature senescence and age-related hearing impairment (ARHI). CMS121 inhibits oxytosis/ferroptosis by targeting fatty acid synthase. The aim of our study was to determine whether CMS121 is protective against ARHI in SAMP8 mice. Auditory brainstem responses (ABRs) were used to assess baseline hearing in sixteen 4-week-old female SAMP8 mice, which were divided into two cohorts. The control group was fed a vehicle diet, while the experimental group was fed a diet containing CMS121. ABRs were measured until 13 weeks of age. Cochlear immunohistochemistry was performed to analyze the number of paired ribbon-receptor synapses per inner hair cell (IHC). Descriptive statistics are provided with mean ± SEM. Two-sample *t*-tests were performed to compare hearing thresholds and paired synapse count across the two groups, with alpha = 0.05. Baseline hearing thresholds in the control group were statistically similar to those of the CMS121 group. At 13 weeks of age, the control group had significantly worse hearing thresholds at 12 kHz (56.5 vs. 39.8, *p* = 0.044) and 16 kHz (64.8 vs. 43.8, *p* = 0.040) compared to the CMS121 group. Immunohistochemistry showed a significantly lower synapse count per IHC in the control group (15.7) compared to the CMS121 group (18.4), *p* = 0.014. Our study shows a significant reduction in ABR threshold shifts and increased preservation of IHC ribbon synapses in the mid-range frequencies among mice treated with CMS121 compared to untreated mice.

## Introduction

Age-related hearing impairment (ARHI) is the most common cause of hearing loss. It has been shown to be associated with cognitive decline, dementia, and depression and results in an estimated annual economic burden of over $3 billion in medical expenditures (Deal et al. 2017, 2018; Lin and Albert 2014). Although the use of hearing aids and/or cochlear implants may improve these associated conditions, ARHI remains significantly undertreated, and to date, there are no targeted therapies (Deal et al. 2018).

### SAMP8 Mouse Model

Laboratory mouse models are invaluable resources for hearing research (Ohlemiller et al. 2016), as mouse and human ears are functionally and genetically homologous. As age is the greatest risk factor for hearing loss, mouse models of aging such as the senescence-accelerated mouse prone (SAMP) strains, which were derived from AKR/J mice and selected for senescence acceleration (Takeda et al. 1981), are excellent resources for the study of ARHI.

Specifically, the senescence-accelerated mouse prone 8 (SAMP8) strain has been shown to develop premature ARHI (Menardo et al. 2012; Peixoto Pinheiro et al. 2021) and exhibit early increased oxidative stress (Benkafadar et al. 2019), which leads to chronic inflammation and the triggering of cell death, resulting in premature ARHI and senescence (Menardo et al. 2012). Functional studies using auditory brainstem response (ABR) have shown SAMP8 mice to be a fast and robust model for the study of aging-related diseases such as ARHI (Marie et al. 2017), and thus, this mouse model provides opportunities to investigate potential ARHI drug candidates.

### The Oxytosis/Ferroptosis Pathway and CMS121

Given the common cellular pathways leading to age-related dysfunction in the brain and cochlea, there is a significant precedent to study compounds for their ability to not only improve cognitive function but also attenuate ARHI. For example, EUK-207, a synthetic superoxide dismutase/catalase mimetic which suppresses oxidative stress, has been shown to both decrease age-related cognitive impairment in C57BL/6N mice (Liu et al. 2003; Clausen et al. 2010) and slow down ARHI in SAMP8 mice (Benkafadar et al. 2019). N-acetylcysteine (NAC) is another antioxidant which has been associated with improved memory performance as well as improved hearing in SAMP8 mice (Marie et al. 2018).

In the study of neuronal cell death pathways and their relationships with age-related neurological disease, the oxytosis/ferroptosis pathway, a regulated cell-death pathway involving glutathione depletion, lipoxygenase activation, reactive oxygen species accumulation, and mitochondrial and calcium dysregulation, has emerged as a potential key driver of pathology in neurodegenerative diseases (Lewerenz et al. 2018; Maher et al. 2020). Although oxytosis/ferroptosis has not been widely studied in the field of hearing loss, this pathway has been associated with neurodegeneration of the auditory cortex in ARHI (Chen et al. 2020).

As glutathione depletion is a key step in the oxytosis/ferroptosis pathway, Maher et al. (2020) identified the flavonol fisetin as a compound of interest in the study of neurodegenerative diseases due to its ability to maintain glutathione levels in the presence of oxidative stress (Ishige et al. 2001; Maher 2009). Importantly, fisetin has been shown to enhance memory in normal animals (Maher et al. 2006), APPswe/PS1dE9 transgenic AD mice (Currais et al. 2014), and SAMP8 mice (Currais et al. 2018).

Further pharmacokinetic studies into fisetin derivatives revealed CMS121 as a promising candidate with enhanced neuroprotective activity and good oral bioavailability (Chiruta et al. 2012). CMS121 reduces lipid peroxidation through activation of AMPK and inhibition of fatty acid synthase. It has since been shown to reduce metabolic and gene transcription markers of aging in SAMP8 mouse brains (Currais et al. 2019) and reduce neuroinflammation and cognitive decline in APPswe/PS1dE9 transgenic AD mice (Ates et al. 2020). Given the potent neuroprotective effects of CMS121 in SAMP8 mice in the context of aging, we hypothesized that it would likewise be protective against ARHI in SAMP8 mice. In this study, we investigated the changes in ABR thresholds and suprathreshold wave I amplitudes in SAMP8 mice treated with CMS121 compared to untreated SAMP8 mice. As disruption in ribbon synapses between inner hair cells (IHCs) and auditory nerve fibers (ANFs) is an early pathological change in ARHI (Xiong et al. 2020), we also compared ribbon-receptor synapse counts between treated and untreated groups.

## Materials and Methods

### Experimental Design

#### Animals

The SAMP8 line was originally acquired from Harlan Laboratories (UK) and subsequently bred and housed at the Salk Institute in accordance with the US Public Health Service Guide for Care and Use of Laboratory Animals and protocols approved by The Institutional Care and Use Committee (IACUC) at the Salk Institute. ABR testing and subsequent cochlear harvesting were performed on the SAMP8 mice in accordance with protocols approved by the IACUC at the University of California, San Diego.

The experimental design consisted of sixteen SAMP8 mice divided into two cohorts of eight mice. Both cohorts underwent baseline ABR testing at the age of 4 weeks. The *control group* was then fed a vehicle diet (LabDiet 5015, TestDiet, Richmond, IN), while the *experimental group* was fed a diet with CMS121 (LabDiet 5015 + 200 ppm CMS121, TestDiet, Richmond, IN) (Chiruta et al. 2012). This dose of 200 ppm correlates to about 17 mg/kg/day and was chosen because it had previously been shown to reduce cognitive decline in SAMP8 mice (Currais et al. 2019) as well as APPswe/PS1dE9 transgenic AD Mice (Ates et al. 2020) with no indications of any adverse effects, even after 4 months of treatment. ABR measurements were then repeated at 7, 10, and 13 weeks of age.

#### Auditory Brainstem Response (ABR)

All ABR testing was performed on mice under intraperitoneal anesthesia (ketamine 80–100 mg/kg body weight and xylazine 10 mg/kg body weight), and all efforts were made to minimize suffering. A thermocouple rectal probe was inserted, and mouse body temperature was maintained via a TCAT-2DF temperature controller and the HP-4 M heating plate (Physitemp Instruments Inc., Clifton, NJ). Mice recovered from anesthesia on a heating pad.

ABR testing was performed inside a MAC-1 sound-proof chamber designed by Industrial Acoustics (IAC, Bronx, NY) to eliminate environmental and electrical noise. Auditory stimuli were generated with a data acquisition board from National Instruments (National Instruments Corporation, Austin, TX) and were delivered using an Intelligent Hearing Systems speaker (Intelligent Hearing Systems, Miami, FL) attached to a 0.8 cm long tube that was inserted into the ear canal. Sound pressure was measured through the use of a condenser microphone. Stainless steel electrodes were placed subcutaneously at the vertex of the head and the right mastoid, with a ground electrode at the base of the tail.

Auditory signals were presented to the right ear as tone pips with a rise and fall time of 0.5 ms and total duration of 5 ms at 4, 8, 12, 16, 24, and 32 kHz frequencies. Tone pips were delivered below threshold and increased in 5 dB increments until the maximum volume of 100 dB was reached. Signals were presented at a rate of 30/second. Signals were then sent to an amplifier and then to a sound transducer from Intelligent Hearing Systems. Physiologic responses were recorded at a 20,000 Hz sampling frequency and sent to an 8 channel 150 gain AC/DC headbox and then onto a secondary Synamps signal amplifier of 2500 gain before analysis. Responses were bandpass filtered between 0.3 and 3 kHz. For each stimulus intensity, 512 waveforms were averaged. Hearing threshold was determined by visual inspection of ABR waveforms and defined by the minimum intensity at which a wave I complex was distinguishable. Suprathreshold Wave I amplitudes were measured from peak to following trough at 80 dB SPL for each mouse. For each mouse, only the right ear was tested, as the dosage of anesthesia given only provided sedation for the length of time required to test one ear, and repeated dosage was avoided to reduce morbidity/mortality.

#### Cochlear Whole Mount Immunolabeling

Following the final round of ABR measurements, the anesthetized mice were intracardially perfused and their harvested cochleae were post-fixed with 4% paraformaldehyde for 1 h for whole-mount functional synapses analysis. Fixed samples were rinsed extensively in phosphate-buffered saline (PBS) and dissected under a microscope; the Organ of Corti from each cochlea was isolated and divided into apical, middle, and basal segments.

The specimens were thoroughly washed with PBS and blocked with 10% goat serum for 1 h at room temperature. Tissues were then incubated at 37 °C with the following primary antibodies: monoclonal mouse anti-carboxyl-terminal binding protein 2 (CtBP2) IgG1 at 1:200 (612,044; BD Biosciences), monoclonal mouse anti-GluR2 IgG2a at 1:1000 (MAB397; Millipore), and polyclonal rabbit anti-myosin VIIa at 1:200 (25–6790; Proteus Biosciences). The following day, after further PBS washes, the tissues were incubated with appropriate conjugated secondary antibodies at a concentration of 1:1000 for 1 h in darkness at room temperature. The samples were then thoroughly washed one final time and mounted on slides using ProLong Glass antifade mount and left to dry for at least 24 h prior to image acquisition.

Frequency regions corresponding to 16 kHz were located based on the place-frequency map from Müller et al. (2005). The immunofluorescence-labeled whole-mount segments were then imaged on a Zeiss 880 LSM Airyscan confocal microscope (Carl Zeiss, Oberkochen, Germany). Images for synapse quantification were acquired with a Plan-Apochromat 63x/1.4 Oil DIC M27 objective, with 42.5 nm *X*–*Y* pixel size and 185 nm *Z-*step size; laser powers used were HeNe633 (49.8 uW), DPSS 561–10 (268.83 uW), Diode 405–30 (122.49 uW), and ArgonRemote (82.47 uW). Images for hair cell counts were acquired with a Plan-Apochromat 10x/0.45 M27 objective, with 171.6 nm *X*–*Y* pixel size and 695 nm *Z*-step size; laser powers used were HeNe633 (49.8 uW), DPSS 561–10 (268.83 uW), Diode 405–30 (122.49 uW), and ArgonRemote (76.69 uW). After acquisition, the images were Airyscan processed using automatic default settings, and the number of punctae corresponding to synaptic ribbons and glutamate receptors per four inner hair cells (IHCs) were blindly counted to determine average number of functional synapses per IHC for each mouse.

### Statistical Analysis

Descriptive statistics are provided with mean ± SEM (standard error of the mean). Welch’s *t*-tests were performed to compare hearing thresholds, wave I amplitudes, and paired synapse counts across the two groups, with alpha = 0.05. Statistical analysis was performed using the R environment for statistical computing (R Core Team 2021).

## Results

ABR thresholds were examined at 4, 8, 12, 16, 24, and 32 kHz. Baseline hearing thresholds (pre-treatment) were obtained for eight 4-week-old mice assigned to the control group (*n* = 8) and eight 4-week-old mice assigned to the CMS121 group (*n* = 8). Following the first ABR measurement, two of the mice from the control group died. We determined that the average baseline thresholds among the remaining six mice were not significantly different from that of the original eight mice in the control group.

**Fig. 1:**
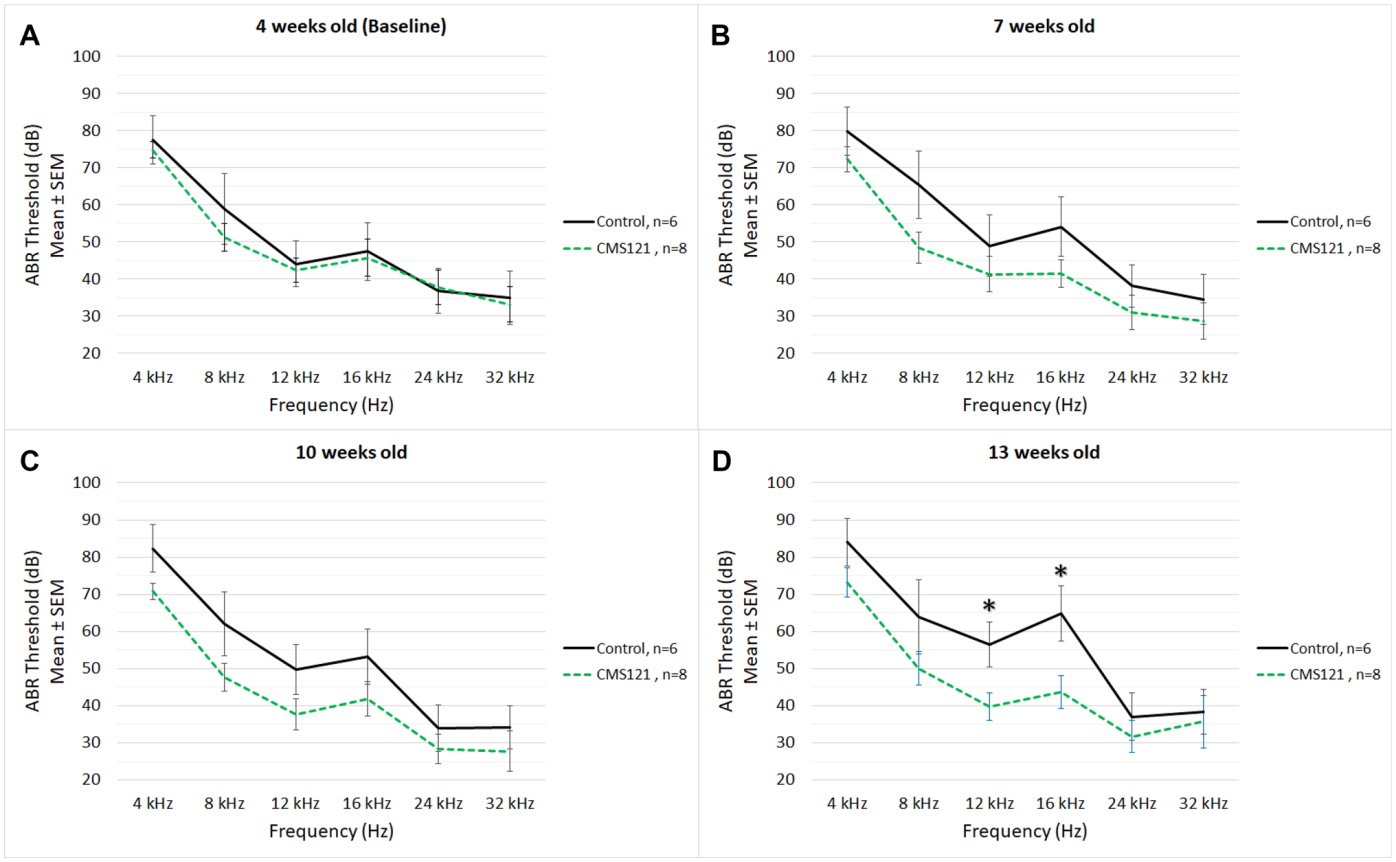
Auditory brainstem response (ABR) thresholds of untreated SAMP8 mice (black solid line, *n* = 6) and CMS121-treated SAMP8 mice (green dashed line, *n* = 8) at 4, 8, 12, 16, 24, and 32 kHz at the indicated ages: 4 weeks (baseline, **A**), 7 weeks (**B**), 10 weeks (**C**), and 13 weeks (**D**). Data are shown as mean ± SEM (standard error of the mean). Significance was set at *p < .05

Baseline hearing thresholds of the mice assigned to the control group (*n* = 6) were similar compared to those assigned to the CMS121 group (*n* = 8) at 4 weeks of age (Fig. 1A). There was no significant difference in ABR thresholds at any frequency between the control group and CMS121 group at 7 and 10 weeks of age (Fig. 1B, C) although there was an age-dependent trend towards higher hearing thresholds in the control group. At 13 weeks, the control group had significantly higher hearing thresholds compared to the CMS121 group at 12 kHz (56.5 vs. 39.8, *t*(8.7) = −2.35, *p* = 0.045) and 16 kHz (64.8 vs. 43.8, *t*(8.4) = −2.45, *p* = 0.039) (Fig. 1D).

**Fig. 2:**
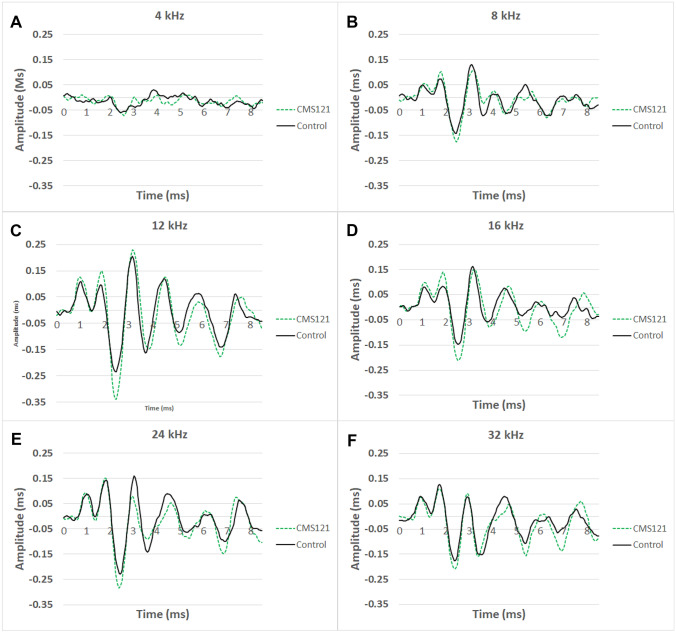
ABR waveforms at 80 dB, averaged across untreated SAMP8 mice (black solid line, *n* = 6) and CMS121-treated SAMP8 mice (green dashed line, *n* = 8). Mean wave 1 amplitude for each cohort is indicated on each graph. Significance was set at *p < .05. There was no significant difference in wave 1 amplitude across the two groups at any frequency

We then evaluated ABR waveforms at 80 dB sound pressure level (SPL) across all mice at 13 weeks of age (when there was a significant difference in hearing thresholds) by measuring the wave I amplitude (peak to following trough) for each mouse. On average, the wave I amplitudes (mean (SD)) across all six frequencies were not significantly different between the mice in the control group vs. the CMS121 group: 0.06 (0.10) vs. 0.08 (0.07) at 4 kHz, *p* = 0.67; 0.22 (0.26) vs. 0.28 (0.19) at 8 kHz, *p* = 0.61; 0.33 (0.26) vs. 0.49 (0.16) at 12 kHz, *p* = 0.18; 0.23 (0.21) vs. 0.35 (0.23) at 16 kHz, *p* = 0.34; 0.37 (0.13) vs. 0.43 (0.15) at 24 kHz, *p* = 0.39; 0.30 (0.12) vs. 0.32 (0.15) P at 32 kHz, *p* = 0.83 (Fig. 2). Representative power analysis of the 16 kHz data (pooled SD = 0.22) shows that 53 mice per group would be needed to detect 0.12 wave 1 amplitude difference between groups using alpha = 0.05 and desired power = 0.80.

At each ABR testing timepoint, mouse weights were also collected. There was no significant difference between the control and CMS121 groups’ baseline weights at 4 weeks of age (17.2 vs. 18.4 g, *p* = 0.19). At 13 weeks, the control group weight was significantly less compared to the CMS121 group (24.5 vs. 28.4 g, *p* = 0.040) (not shown).

**Fig. 3:**
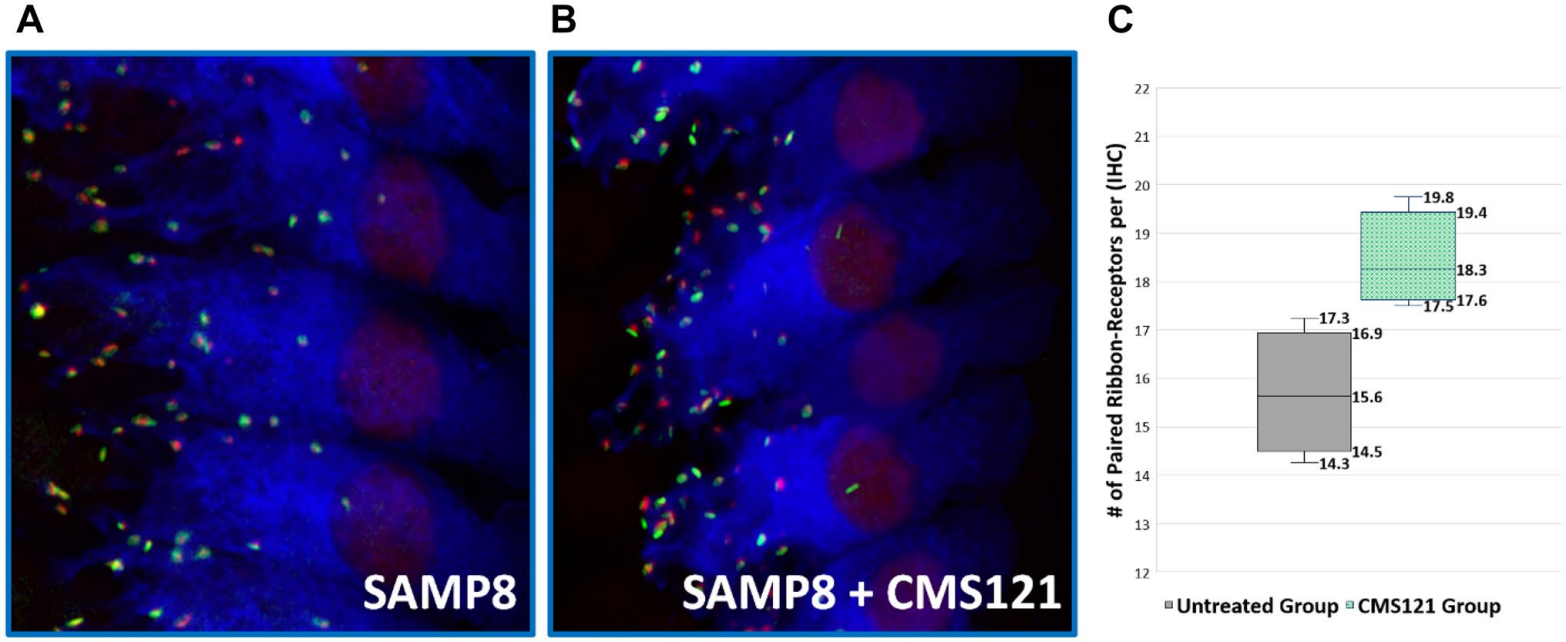
Representative images of synaptic immunolabeling for the 16 kHz cochlear region of an untreated SAMP8 mouse (**A**) and a CMS121-treated SAMP8 mouse (**B**). CtBP2 labeled red and GluR2 labeled green to visualize the pre-synaptic ribbon protein and post-synaptic glutamate receptor, respectively. Paired ribbon-receptors were blindly counted across four inner hair cells (IHCs) in each mouse to determine the average number of ribbon synapse counts per IHC, which was compared between the untreated SAMP8 mice (gray) and CMS121-treated SAMP8 mice (green). Significance was set at *p < .05 (**C**). There was a significantly lower synapse count per IHC in the untreated group (15.6) compared to the CMS121-treated group (18.3), *p* = 0.014

Representative confocal imaging of inner hair cells from the control group (A) and CMS121 group (B) are shown in Fig. 3A, B. Immunohistochemistry of cochleae from both conditions following the final round of ABR measurements showed a significantly lower synapse count per IHC in the untreated group (15.6) compared to the CMS121-treated group (18.3), *p* = 0.014 (Fig. 3C).

## Discussion

In the USA, hearing loss is one of the key contributors to increases in chronic disability (Murray et al. 2013). Estimates suggest that approximately two-thirds of people over the age of 70 in the USA experience ARHI (Bainbridge and Wallhagen 2014). Globally, it is projected to be among the top ten causes of disease burden in high- and middle-income countries by the year 2030. Hearing loss is associated with numerous adverse social and health-related effects (Arlinger 2003). In particular, hearing loss is independently associated with dementia (Lin et al. 2011; Lin and Albert 2014) and is furthermore the strongest potentially modifiable risk factor for developing dementia (Livingston et al. 2017).

The SAMP8 mouse model has been shown to be a robust model of ARHI (Marie et al. 2017), wherein oxidative stress, altered levels of antioxidant enzymes, and decreased activity of complexes I, II, and IV lead to chronic inflammation and triggering of cell death pathways that ultimately result in the degeneration of outer hair cells, spiral ganglion neurons, stria vascularis, and inner hair cells, mimicking human ARHI (Menardo et al. 2012).

To date, there exist no pharmacologic agents approved for the treatment or prevention of ARHI. The fisetin derivative CMS121 is a promising drug candidate that has been shown to improve cognitive function in SAMP8 mice with symptoms of dementia (Chiruta et al. 2012; Currais et al. 2018). Although the mechanism of action of CMS121 is still under study, it in part shows neuroprotective activity against insults such as oxytosis/ferroptosis by partially inhibiting the lipid biosynthetic enzyme, fatty acid synthase, thereby decreasing lipid peroxidation, and protecting cells against increased oxidative stress and inflammation (Ates et al. 2020; Maher et al. 2020). It has also been shown to preserve brain mitochondrial gene expression via inhibition of acetyl-CoA carboxylase 1 (ACC1) and the maintenance of high levels of the central mitochondrial metabolite acetyl-Coenzyme A (acetyl-CoA) (Currais et al. 2019). In prior studies, CMS121 has been shown to delay molecular markers of aging and cognitive decline in SAMP8 mice (Currais et al. 2019). Given that oxidative stress, lipid peroxidation, mitochondrial dysfunction, and cell death in the cochlea are also thought to play a key role in ARHI (Someya et al. 2009; Fujimoto and Yamasoba 2014), this study is aimed at evaluating CMS121 as a potential drug candidate for the prevention of ARHI in SAMP8 mice.

### CMS121-Treated Mice Exhibited Attenuation of ARHI in the Mid-Range Frequencies

Our study followed functional measurements of hearing in untreated SAMP8 mice versus CMS121-treated SAMP8 mice. SAMP8 mice have been shown to develop progressive age-related ABR threshold increases characteristic of ARHI (Marie et al. 2017; Peixoto Pinheiro et al. 2021). Among the untreated mice in our study, we observed the expected progressive ABR threshold increases from 4 to 13 weeks of age. Among the CMS121-treated mice, we observed a significant attenuation in ARHI resulting in stable ABR thresholds from 4 to 13 weeks. At 13 weeks of age, the CMS121-treated mice on average had significantly lower ABR thresholds in the mid-range frequencies (12 and 16 kHz) compared to the untreated mice, who experienced progressive threshold increases at these mid-range frequencies. Although different mouse strains have been shown to exhibit different frequency sensitivities to hearing loss (Zheng et al. 1999), these mid-frequencies are where mice are typically most sensitive to sound (Reynolds et al. 2010). This suggests that the benefit of hearing impairment attenuation at these frequencies may be especially advantageous. The mechanisms underlying hair cell death and synaptic ribbon loss with age are still poorly understood in both mice and humans. It is notable that ABR thresholds for 24 kHz and 32 kHz were not significantly improved after CMS121 treatment, possibly the result of confounding genetic contributions from the strain-related hearing loss and/or additional age-related vulnerabilities of the basal-most cochlear regions.

### CMS121-Treated Mice Retained Higher Numbers of IHC/ANF Synapses

In recent years, research has suggested that auditory aging results not only in audiometric threshold elevations following hair cell loss but also in “hidden” hearing loss including perceptual difficulties in understanding speech in complex sound environments in the setting of stable audiometric thresholds (Liberman 2017; Liberman and Kujawa 2017). In ARHI, it has been shown that degeneration of cochlear synapses precedes both hair cell loss and threshold elevation (Sergeyenko et al. 2013) and that the synapses between IHCs and ANFs in the aging cochlea are the most vulnerable elements, not the hair cells (Kujawa and Liberman 2015). We examined the integrity of IHC/ANF synapses in this study in two ways.

#### Wave I Amplitude

The ABR waveform comprises several peaks and troughs within the first ~ 10 ms after acoustic stimulus onset (Akil et al. 2016; Young et al. 2021). Wave I occurs around 2 ms and represents the summated response from the spiral ganglion and auditory nerve (Akil et al. 2016). Wave I suprathreshold amplitudes have been associated with significant differences in functional synaptic ribbon counts (Boussaty et al. 2020). In this study, we did not observe any significant differences in suprathreshold wave I amplitudes between the CMS121-treated and untreated mice, although there were weak trends towards significance in the mid-range frequencies which may be further developed in future studies with larger samples of mice. Our preliminary power analysis suggests that sample sizes of 50 mice per group would be necessary to reach significance for the wave 1 comparisons.

#### Synaptic Immunolabeling

At the conclusion of the ABR measurement series, we also directly examined functional synaptic counts (paired ribbon-receptors puncta) as a measurement of hidden hearing loss and as another measurement of cochlear aging. We observed that at 13 weeks, the CMS121-treated SAMP8 mice had higher numbers of functional synapses between mid-frequency IHCs compared to untreated SAMP8 mice. Disruptions in functional ribbon synapses has been shown to reduce hair cell ability to transmit signals with temporal precision (Jean et al. 2018) and thus impair the neural encoding of acoustic temporal cues essential for speech comprehension (Moser et al. 2013). This finding further reinforces the functional ABR threshold differences at 16 kHz by showing the disruption in ribbon synapses in aging SAMP8 mice and how that disruption was attenuated in SAMP8 mice treated with CMS121.

In concert, these findings suggest that treatment with CMS121 is associated not only with improvement in audiometric signs of hearing impairment (i.e., increased ABR thresholds in untreated mice) but also improvement in impaired ribbon synapses which may be associated with synaptopathy and decreased ability to comprehend auditory stimuli.

### Limitations

#### Sample Size

Although our sample size was adequately powered for identifying differences in ABR thresholds between groups, we were limited in the number of available age-matched SAMP8 strain mice for the study so this study would still benefit from replication with larger numbers of animals, including male mice. The analysis of secondary outcomes such as wave I amplitude differences would yield more robust results if replicated with a larger sample size.

#### Mouse Longevity

By their nature as senescence-accelerated animals, we found that SAMP8 mice are less tolerant of anesthesia compared to other inbred models, as suggested by the two animals that expired prematurely following the first round of ABRs. While it would have been preferable to continue the experiment for several more weeks, we ended the final round of ABRs at 13 weeks to avoid further repeated stressors. Furthermore, along the same lines, we chose to start with young mice due to the concern that starting with older mice would not yield a sufficient time frame for testing given poor longevity after serial rounds of anesthesia for testing.

#### Mouse Weights

Although there was initially no significant difference in mouse weights between groups, at 13 weeks, the control group weighed significantly less than the group treated with CMS121. It is unclear to what extent this difference in weight is natural variance that may exist between different SAMP8 mice, versus an effect of CMS121 itself.

#### Unilateral ABRs

For each mouse, only the right ear was tested to reduce anesthesia-associated morbidity/mortality. Although ARHI often presents bilaterally, threshold shifts may not necessarily be symmetric. However, we standardized by using only the right ear for all animals, so we do not expect this to influence any between-group comparisons.

## Conclusion

In summary, these findings support expanding the scope of current research on CMS121 to further investigate the promising role of this compound as a protective agent against ARHI. Future studies should also consider testing CMS121 as a treatment for ARHI after it has already occurred. CMS121 is currently finishing up a phase 1 clinical trial for safety in healthy, young humans (NCT05318040) and thus is poised to be tested for efficacy in age-related human diseases such as ARHI.

## Data Availability

The data that support the findings of this study are available from the corresponding author, RAF, upon reasonable request.

## Abbreviations

ABR: auditory brainstem response
AD: Alzheimer’s disease
ANF: auditory nerve fiber
ARHI: age-related hearing impairment
DPOAE: distortion product otoacoustic emissions
IHC: inner hair cell
SAMP8: senescence-accelerated mouse prone 8
SEM: standard error of the mean
